# Logical validation and evaluation of practical feasibility for the SCRuM (School Clinical Rugby Measure) test battery developed for young adolescent rugby players in a resource-constrained environment

**DOI:** 10.1371/journal.pone.0207307

**Published:** 2018-11-20

**Authors:** Matthew Chiwaridzo, Danai Chandahwa, Sander Oorschot, Cathrine Tadyanemhandu, Jermaine M. Dambi, Gillian Ferguson, Bouwien C. M. Smits-Engelsman

**Affiliations:** 1 Department of Health and Rehabilitation Sciences, Division of Physiotherapy, Faculty of Health Sciences, University of Cape Town, Cape Town, South Africa; 2 Rehabilitation Department, University of Zimbabwe, College of Health Sciences, Avondale, Harare, Zimbabwe; 3 Department of Rehabilitation, Academic Medical Center, University of Amsterdam, Amsterdam, Netherlands; University of Essex, UNITED KINGDOM

## Abstract

There is a growing impetus towards usage of test batteries in talent identification (TID) programmes in rugby. Consequently, there are many test batteries in existence profiling anthropometric, physiological characteristics and rugby-specific skills. There is no consensus in the literature on the constituent variables and corresponding tests required to inform TID programs. Following development of a new test battery called the SCRuM (School Clinical Rugby Measure), this study aimed at establishing face, logical validity and practical feasibility of included tests. The test battery, initially comprised of 23 items, had its face and logical validity evaluated by five (5) adolescent rugby coaches and 20 rugby experts, respectively. Logical validation was conducted in two questionnaire-based rounds with Content Validity Index (I-CVI) calculated for each variable. Subsequently, a cross-sectional study targeting 30 local rugby coaches was conducted to determine the perceived practical feasibility of each test item. The results showed excellent I-CVI (>0.78) for 17 variables (speed, weight, height and skin fold measures, repeated high-intensity exercise performance ability, prolonged high-intensity intermittent running ability, change of direction speed, anaerobic capacity, lower-and upper body muscular power and strength, muscular flexibility, reactive agility, passing for accuracy, tackling proficiency, and catching). However, three tests, namely, Reactive Agility, One Repetition Maximum Back Squat and One Repetition Maximum Bench Press had low test-feasibility indices (T-FI< 35) suggesting practicality concerns with implementation in the Zimbabwean context. Thus, these findings suggest the need for substitution or development of new practically feasible tests for upper-and lower body muscular strength and reactive agility.

## Introduction

Rugby union (rugby) is a popular sport even in countries hardly known for competitive rugby such as Zimbabwe [1, 2]. With the advent and subsequent global spread of professionalism in rugby since 1995 [3], an increased number of adolescents are participating either professionally or otherwise in this physically demanding collision sport worldwide [4]. Possibly, with continued professionalism and increased demand for young competent rugby players with potential to become successful future elite athletes, the number of young players is likely to increase and also efforts directed towards identifying and recruiting young rugby players will heighten universally [3–6]. Central to the process of talent identification (TID) and recruitment of young rugby talent is the development and usage of screening test batteries composed of variables reflecting the key requirements of rugby and practically feasible tests with acceptable psychometric properties [7–14]. Currently, there is no general consensus in the literature on the ideal constituent variables and the corresponding tests that should be included in test batteries designed to inform TID. Consequently, existing test batteries are varied in composition having dissimilar tests assessing similar construct variables. This is a significant shortcoming when comparison data profiling young rugby players is needed.

Despite the complexity of TID programs, the cardinal focus of such programs should be on the objective assessment of key and minimal requirements of the sport of rugby in potential players [5] utilising standardised test batteries. This implies that it is the key requirements of rugby which should provide a theoretical framework underpinning the selection of component variables for inclusion during test battery development [15]. However, in order to understand the key attributes, qualities or skills needed in rugby and, concomitantly, the variables to include in screening test batteries, knowledge of the physical demands of rugby is essential. This knowledge helps in understanding the locomotor and non-locomotor patterns common in rugby [16, 17] and, consequently, facilitates development of test batteries that replicates match demands. Furthermore, alternative approaches in understanding variables to include in test batteries involves establishing qualities, attributes or skills differentiating rugby players by levels of competition or related to match performance [18]. Identified qualities, attributes or skills with high discriminative ability and/or are associated with effective playing performance may then be incorporated in test batteries, as they potentially indicate important attributes required by rugby players.

Studies utilising Time Motion Analysis (TMA) and Global Positioning System (GPS) have shown that rugby is a dynamic, intermittent, and highly demanding physical sport [17, 19–29]. It is known that regardless of level of competition, rugby players spend 79% to 94% of match time in low-intensity activities (LIA), interrupted briefly by moderate-to-high intensity running or non-running activities such as striding, cruising, sprinting, static exertions (rucking, mauling, scrummaging), and tackling [17, 25–33]. It is these short, high-intensity activities (HIA) that are most crucial in rugby, possibly determining the outcome of rugby matches in terms which team win or lose a particular rugby match. Accordingly, understanding the key characteristics and skills needed to repeatedly perform these intense activities for the duration of a rugby match should have important implications in test battery design. Additionally, static exertions and power-based tasks such as tackling occur throughout the game of rugby and require high levels of upper-and-lower body muscular strength and power [17, 34]. As such, numerous studies have documented evidence of the relationship between these physiological characteristics and future career success or team selection [3, 34–38]. Therefore, it seems logical to include measurements of upper-and-lower body muscular strength and power in test batteries designed for screening potential rugby talent in TID programs given and also for general profiling of rugby players given the importance of these characteristics in rugby.

Literature has also shown that sprinting efforts over short and long distances are key movement patterns commonly observed in rugby for both forward and back players [25, 32, 33, 39–43]. Therefore, testing of speed is important and should be over varied distances reflecting the requirements for the different positions. With repeated efforts, both sprinting and static exertions require some degree of endurance considering 70 or 80 minutes of match play [17, 31, 44–46]. Thus, the ability to perform repeated HIA is essential and potentially important to screen for when identifying talent [47, 48]. Specifically, repeated sprinting ability (RSA) and repeated high-intensity exercise (RHIE) performance ability should be important components in test batteries [34, 47–51]. Smart et al [51] showed an association between speed and RSA with tries scored in a match, suggesting the importance of these characteristics in relation to match performance. Additionally, Gabbett [48] reported moderate to large effect size differences between the starters and non-starters rugby league players for speed, change of direction speed (CODS) and aerobic capacity, further illustrating the importance of these characteristics.

Pienaar et al [52] assembled a test battery measuring 21 anthropometric variables, eight (8) motor and physical abilities and six (6) games skills for identifying young South African rugby players. Also, test batteries utilised by Van Gent and Spamer [53] and Spamer and De la Port [54] for the same population had a similar multi-dimensionality. However, critical appraisal of these test batteries showed that the rationale for inclusion of the test items was seldom provided in the content of the articles and several variables were missing which would have given sufficient logical validity to the test batteries that are designed particularly for profiling anthropometric, physical, physiological abilities and rugby-specific skills among young players. Variably, the batteries excluded tests for tackling, reactive agility, RHIE performance ability, repeated effort ability (REA), (an)aerobic capacity, and lower-body muscular strength and power which probably emphasise the intermittency and physical nature of rugby. It is, therefore, imperative when developing test batteries for young adolescent rugby players to include test items that logically and comprehensively reflects the demand components of the game and has reliable, valid and feasible tests for the context it is to be implemented. Test batteries that are logically validated to the needs of the young rugby players containing also practically feasible tests are more likely to be relevant for use in screening or talent recruitment programs and to be implemented by the intended users such as coaches, strength and conditioning experts and sports scientists. Such test batteries can be consistently used to determine players’ competency levels, TID, creating a profile of each individual athlete, tracking progress over time and also evaluating the effectiveness of interventions [12]. Therefore in an attempt to comprehensively understand the key physical, physiological and skill-based needs of young male adolescent rugby players between the ages of Under 16 and Under 20, based on shared consensus among rugby experts, this study evaluated the logical validity of the test variables included in a newly-developed test battery called the School Clinical Rugby Measure (SCRuM) and, further evaluated the practical feasibility of each corresponding test in the test battery in the Zimbabwean context. In the broader context of the large doctoral study in which this study was part of, the test battery was developed with the ultimate aim of determining anthropometric, physical or physiological characteristics and rugby-specific game skills discriminating young (U16-U20) Zimbabwean male rugby players by level of competition.

## Material and methods

### Test battery development

The present study was conducted as part of a large multi-phased study (Table 1). Briefly, Phase I entailed development of the first version of the SCRuM test battery through a three-part process (Table 1). The process was informed by literature recommendations for instrument or test battery development [55–58]. However, the actual selection of the candidate items and corresponding tests considered interplay of several factors such as:

1. The physical, motor or physiological characteristics and rugby-specific technical game skills identified to be commonly assessed in the literature.
2. The tests frequently used for the assessment of each of the identified construct in rugby and other related intermittent sports such as rugby league. However, tests found specifically developed for rugby players were preferentially selected for inclusion over generic tests for the corresponding variable.
3. The qualities, attributes or skills local rugby coaches perceived to be important in defining a good rugby player and are important for consideration during talent recruitment in TID programs.
4. The tests known and frequently used by rugby coaches in the local context during training or for assessment of players.
5. The availability of acceptable psychometric or measurement properties (reliability, validity and responsiveness) for the test based on the Consensus-based Standards for the Selection of health Measurement Instruments (COSMIN) checklist.
6. The level of evidence for the test based on “best evidence synthesis” of the psychometric properties based on the the quality criteria for rating of measurement properties provided by Terwee et al [59].

**Table 1:** Methodological stages used to develop and validate the SCRuM test battery.

Phase	Part	Aim	Methodology
Phase 1	Part I	Determined what is known about the key requirements of rugby specifically targeting anthropometric, physical or physiological characteristics and rugby specific game skills in literature.	Narrative literature review
	Part II	Explored perceptions of rugby coaches on the key attributes or qualities and game skills needed in rugby and should be incorporated in test batteries for TID programs. This part also sought commonly used test(s) for the identified attributes and skills used in the local context	A qualitative study
	Part III	Determined frequently assessed physical or physiological characteristics and rugby-specific game skills and their corresponding tests in literature and evaluate the psychometric properties of each identified tests per construct [10, 11].	Systematic review
Phase 2	Part I	Determined face validity of the first version of SCRuM test battery.	Face validation study using key informants
	Part II	Determined the logical validity of the second version of the SCRuM test battery. Logical validity was assessed in two rounds, engendering the third and fourth version.	Logical validation study using rugby experts
	Part III	Assessed the practical feasibility of the test items in the fourth version of the SCRuM test battery, engendering the fifth version of the SCRuM test battery.	Cross-sectional descriptive study using local rugby coaches
Phase 3	Part I	Assessment of the test-retest reliability of the fifth version of the SCRuM test battery, engendering the sixth version if there are changes to the content of the fourth version.	Test-retest reliability study
	Part II	Assessment of the construct (discriminative) validity of the sixth version of the SCRuM test battery engendering the final version of the SCRuM test battery with tests able to discriminate young male rugby players by level of competition	Construct validation study

The authors (MC, BSE, SO) formed the working group that selected the test items for the SCRuM test battery largely guided by the ultimate purpose of the test battery and the factors alluded above.

S1 Table shows the first version of the SCRuM test battery and the rationale for choosing each included test. Following development, the test battery was subsequently evaluated for face and logical validity and practical feasibility. This is the part described in this paper. For ease of understanding, the present study was separated into three parts (Part I, II, III) and describes the methods and results for each of these parts.

### Part 1: Face validation of the test battery

Although face validity is not considered an active measure of validity, it yields subjective but important preliminary information on whether study instruments measure what they purport to measure [60]. For the present study, face validity was considered as the extent to which the SCRuM test battery appeared to have component variables measuring the following pre-selected domain constructs: anthropometric, physical or physiological and rugby-specific game skills. Moreover, each test was assessed considering the degree to which it “looked” to be measuring the corresponding variable [61]. The study targeted key informant coaches coaching first team male rugby players from schools in the “elite” Super Eight Schools Rugby League (SESRL) and “sub-elite” Co-Educational Schools Rugby League (CESRL [2] based in Harare, Zimbabwe. The SESRL and CESRL represent the most competitive domestic high school rugby leagues in the country. A researcher-developed questionnaire listed all the SCRuM variables, corresponding tests and details of the test procedure. Participants rated each test based on a Likert scale (1 = strongly disagree, 2 = disagree, 3 = neutral, 4 = agree, 5 = strongly agree) depending on whether the test appeared to be measuring the corresponding variable. Arbitrarily, at least 50% of the respondents had to agree or strongly agree for a test to be considered as having face validity. The questionnaire also elicited qualitative comments for any test item judged 1, 2 and 3. Respondents also had to provide a comment on whether the test battery adequately reflected a compilation of anthropometric, physical or physiological characteristics and rugby-specific game skills. Ethical approval was obtained from the Human Research Ethics Committee at the University of Cape Town (ref: 016/2016) where the lead author is registered as an international doctoral student in the School of Health and Rehabilitation Sciences in the Division of Physiotherapy. In addition, ethical approval was subsequently sought and obtained from the Medical Research Council of Zimbabwe (ref: MRCZ/A/2070), since the study was conducted in Zimbabwe. Also ethical clearance was sought and obtained from the Joint Research Ethics Committee for the University of Zimbabwe, College of Health Sciences and Parirenyatwa Group of Hospitals (JREC ref 418/17), since part of the data was used by one of the co-authors (DC) as part of her undergraduate research project in physiotherapy in the Department of Rehabilitation. Identified rugby coaches provided written informed consent prior to data collection.

### Results

Five (5) high school male coaches, with the median age of 45 years, volunteered to participate in the study (Table 2). Overall, the coaches endorsed the face validity of the SCRuM test battery. However, four of the respondents felt that the Multistage Fitness Test (MSFT) measuring maximal aerobic power was a “duplication” of the Yo-Yo Intermittent Recovery Level I (Yo-Yo IRL1) Test measuring PHIIRA/endurance.

**Table 2:** Face validation results of the newly- assembled SCRuM test battery (N = 5).

SCRuM variable item	Corresponding test	Likert scale responses					Decision to include/exclude
		S. Disagree, *n*	Disagree, *n*	Neutral, *n*	Agree, *n*	S. Agree, *n*	Final Decision
Speed	5m, 10m, 20m, 40m speed test	0	0	0	0	5	Included
RSA	Rugby-specific repeated speed (RS^2^) test	0	0	0	3	2	Included
REA	REA test	0	0	0	4	1	Included
RHIE performance	RHIE performance test	0	0	0	3	2	Included
PHIIRA/endurance	Yo-yo intermittent recovery level 1 test	1	1	0	2	1	Included
Maximal aerobic power	Multistage fitness test	1	3	0	1	0	**Excluded**
Anaerobic capacity	Triple 120m shuttle test	1	0	0	3	1	Included
CODS/agility	L-run test	0	0	0	0	5	Included
LB muscular power	Vertical jump test	0	0	0	3	2	Included
LB muscular strength	One repetition maximum back squat test	0	0	0	1	4	Included
UB muscular power	2kg medicine ball chest throw test	0	0	0	2	3	Included
UB muscular strength	One repetition maximum bench press	0	0	0	3	2	Included
UB muscular endurance	Flexed arm hang	0	0	0	5	0	Included
Abdominal endurance	60seconds sit up test	0	0	0	4	1	Included
Reactive agility	Reactive agility test	0	0	0	5	0	Included
Tackling	Tackling proficiency test	0	0	0	1	4	Included
Catching	Running and catching test	0	0	0	4	1	Included
Kicking	Kicking for distance test	0	1	0	4	0	Included
Passing for distance	Passing for distance test	0	0	0	2	3	Included
Passing for accuracy	Passing for accuracy test	0	0	0	2	3	Included

Anthropometric measures and body composition (skin folds) were omitted in the tables. All coaches agreed to strongly agree for inclusion for those variables; Repeated Sprinting Ability-RSA; PHIIRA-prolonged high intensity intermittent running ability; S.Disagre-Strongly Disagree; S.Agree-Strongly Agree; UB-Upper body; LB-Lower body; REA-repeated effort ability

### Part 2: Logical validity

After the preliminary face validation using coaches identified as key informants, the test battery was subjected to detailed evaluation of its content using rugby experts. Although the term content validity is commonly applied for questionnaires [62], in the context of performance measures the term addresses questions such as “how well a specific test measures what it intends to measure?” or “do the items included in the test cover the entirety of those relevant to assessing a particular skill outcome measure?” [13]. Terms such as logical or definitional validity have also been used interchangeably with content validity [58]. It appears, however, that logical validity is often applied for sports-based tests [8, 55, 63, 64]. For example, Rikli and Jones [55] described logical validity as the degree to which a test (or a test battery) reflects a defined domain of interest. According to Hendricks et al [8], the fundamental question describing logical validity of a rugby test is “does the test measure a relevant and important aspect of rugby?”

### First round

This first round was designed to establish the logical validity of the 22 variables and their corresponding tests in the SCRuM test battery. The COSMIN checklist provided the definitional guideline for logical validity [61]. Logical validity was established through two rounds of expert consultations. Panellists assessed the relevance of SCRuM test battery items by age, gender and overall purpose of the test battery as per the COSMIN guidelines. The primary objective was to determine component items with acceptable Content Validity Index (CVI). Secondly, the study sought to identify characteristics and their corresponding tests missing in the SCRuM test battery but highly recommended for inclusion by at least half of the participating rugby experts.

International and local rugby experts participated in the study. International experts were selected based on being Professors or PhD holders having at least three publications on rugby. The recruitment of local experts was premised on identifying a representative sample of experts with at least 5 years of coaching or playing or directing or involved in rugby in Zimbabwe. A researcher-developed logical validation instrument was used for data collection. Experts rated the relevance of each SCRuM variable based on a Likert scale as follows: *1 = test not relevant*, *2 = test somewhat relevant*, *3 = test quite relevant*, *4 = test highly relevant* [65]. In addition, experts had to comment for test variables rated 1 or 2, recommend missing variables and corresponding test(s).

#### Procedure

This study was conducted between January and February 2018. Possible candidate names for international experts were obtained from an online document listing “top” 100 experts in sports science [66] and also from reference lists of two systematic reviews conducted by the lead author [10, 11]. This was done to determine the authors frequently publishing on physical characteristics and rugby-specific game skills. So, these two strategies provided the sampling frame for the international experts. However, the actual decision for the selection of international experts was based on consensus agreement among three authors (MC, DC and BSE) considering factors such as (i) availability of active and recent email address, (ii) availability of the expert on Research Gate (a research platform enabling us to evaluate expert publications, academic qualifications and biographs) (iii) availability on social media platforms such as LinkedIn or Twitter (an additional invitational platform for experts when emails “bounce”), and (iv) number of publications pertaining to rugby on PubMed or Google Scholar. Email addresses of selected experts were mainly obtained from journal articles and university webpages. In total, 43 international experts were identified and invited to participate via email. Experts were furnished with the online questionnaire through REDCap (a secure web application for building and managing online surveys). For the local experts, purposive sampling method was used for the recruitment, with participants assisting in identifying others (snowballing). In total, 20 local rugby experts were approached and those who agreed to participate signed the informed consent form.

#### Statistical analysis

Item-Content Validity Index (I-CVI) was computed for each test item as the number of experts giving a rating of either 3 or 4 divided by the total number of experts [67–69]. CVI is the most widely used quantitative approach for the content validation [58, 67]. The adopted cut-off for an acceptable I-CVI was >0.78 [68]. Each test item with I-CVI >0.78 was deemed relevant for inclusion in the test battery. The percentage agreement (the number of test items with an I-CVI of 1.00 divided by the total number of items validated in the test battery, expressed as a percentage) was also calculated to represent the proportion of test items experts deemed highly relevant. Scale level-Content Validity Index (S-CVI) was mathematically computed as an average of all I-CVIs [68]. This represented the overall logical validity of the SCRuM test battery. A second round of validation was needed when the S-CVI/Ave for the test battery was below the acceptable cut-off of 0.90 [68]. In addition, an index of inter-rater agreement adjusting for chance agreement [58] was calculated for each test item as indicated in Larsson et al [67].

#### Results

We invited 63 local and international experts of whom 20 (31.7%) agreed to participate. The eight international experts represented United Kingdom (5), South Africa (1), Australia (1) and New Zealand (1). The experts were either professors or PhD holders in human movement sciences, sports physiotherapy or medicine and with preferential interest in rugby. The length of experience ranged from 13 to 25 years mainly in lecturing and sport science research. Of the 12 local experts, they included two (2) sport scientists, senior rugby coaches, and former Zimbabwe national team rugby players, one (1) current Zimbabwe senior national team rugby player, former Zimbabwe national rugby team coach, former Zimbabwe national rugby team sports director, former Zimbabwe national Under-19 team manager, junior rugby sports director at a local school, and physiotherapist for the Zimbabwe national rugby team. Table 3 summarises the rating of each of the variables in the SCRuM test battery.

**Table 3:** Results for first part of the content validation study.

SCRuM variable	R1	R2	R3	R4	R5	R6	R7	R8	R9	R10	R11	R12	R13	R14	R15	R16	R17	R18	R19	R20
Speed	4	4	4	4	4	4	4	3	4	4	4	4	3	4	4	4	4	4	4	4
RSA	4	4	4	2	4	2	3	3	3	4	4	2	4	4	4	4	4	3	4	3
REA	2	4	2	2	4	4	4	3	1	4	4	2	4	4	4	3	4	3	3	4
RHIE	1	4	1	4	3	4	4	3	4	4	4	4	4	4	4	4	4	3	3	2
PHIIRA/Endurance	4	4	4	3	3	3	4	2	4	4	4	4	3	3	4	4	4	4	4	4
Anaerobic capacity	4	4	4	2	3	4	2	2	2	4	3	3	4	4	4	4	3	4	4	4
CODS/agility	4	4	4	4	4	2	3	3	4	4	4	4	3	4	4	3	3	4	4	3
LB muscular power	4	4	4	4	4	4	4	3	4	4	4	4	4	4	4	4	4	3	4	4
LB muscular strength	4	4	4	4	4	4	4	3	4	4	4	4	4	4	4	4	4	3	3	4
UB muscular strength	4	4	4	4	3	3	4	3	4	4	4	4	4	3	4	4	4	4	4	4
UB muscular power	4	4	4	3	4	3	4	3	4	4	4	4	4	4	3	4	4	3	3	3
UB muscular endurance	4	4	4	2	3	1	3	2	2	4	2	4	3	3	3	4	3	3	4	3
Abdominal endurance	2	4	2	2	3	3	2	1	1	4	2	1	3	4	3	4	3	3	3	3
Reactive agility	4	4	4	3	3	4	3	3	4	4	4	3	4	4	4	3	4	3	4	4
Tackling	4	4	4	4	4	4	4	2	4	4	4	3	4	4	4	4	4	3	4	4
Catching	4	4	4	4	4	4	4	2	4	4	4	4	4	4	4	4	3	4	4	3
Kicking	4	4	4	2	3	2	3	2	1	4	2	4	2	3	3	3	2	4	3	4
Passing for distance	4	4	4	2	4	2	3	2	1	4	2	4	2	4	3	3	2	4	4	4
Passing for accuracy	4	4	4	4	4	4	4	3	3	3	4	4	4	4	3	3	4	4	3	4
Height	4	4	4	4	4	4	4	4	4	4	4	4	4	4	4	4	4	4	4	4
Weight	4	4	4	4	4	4	4	4	4	4	4	4	4	4	4	4	4	4	4	4
Skin folds	4	4	4	4	4	4	4	4	4	4	4	4	4	4	3	4	4	4	4	4

R = Rater; 1 = not relevant, 2 = somewhat relevant, 3 = quite relevant, 4 = highly relevant; Prolonged high intensity intermittent running ability = PHIIRA; Repeated sprinting ability = RSA; Repeated effort ability = REA; Repeated High Intensity Exercise Performance = RHIE; Maximal Aerobic Power = MAP; Change of direction speed = CODS; LB = lower body; UB = upper body.

The calculated I-CVIs and the corresponding modified kappa coefficient values for each test item in the SCRuM test battery are shown in Table 4. The calculated I-CVIs ranged from 0.6 to 1. Overall, the test battery achieved an S-CVI/Ave of 0.86. Ten variables had excellent kappa values (k = 1). Hence, the percentage agreement for the SCRuM test battery was 43.5%. Five (5) variables were excluded and the reasons are shown in Table 4. Thematic analysis of the recommended attributes showed that “muscle flexibility” was highly recommended by 13 (65%) of the experts. However, Sit-and-Reach test was reported as the most commonly used test for the variable. The other attributes and skills recommended for inclusion were: defensive and offensive skills (n = 2), balance (n = 3), anticipatory or reaction skills (n = 3), abdominal strength (n = 2) and ball rucking (n = 1).

**Table 4:** Item-content validity indices for the SCRuM test items.

Scrum variables	Relevant (3 or 4)	Not relevant (1 or 2)	I-CVI	Kappa (k)	Interpretation	rationale for exclusion
Speed	20	0	1	1	Validated	
RSA	17	3	0.85	0.85	Validated	
REA	15	5	0.75	0.75	**Excluded**	It mimics RHIE and is not as important as RHIE
RHIE	17	3	0.85	0.85	Validated	
Endurance/PHIIRA	19	1	0.95	0.95	Validated	
Anaerobic capacity	16	4	0.8	0.8	Validated	
CODS/Agility	19	1	0.95	0.95	Validated	
LB muscular power	20	0	1	1	Validated	
LB muscular strength	20	0	1	1	Validated	
UB muscular power	20	0	1	1	Validated	
UB muscular strength	20	0	1	1	Validated	
UB muscular endurance	15	5	0.75	0.75	**Excluded**	Not common and relevant in rugby, indirectly assessed by other variables
Abdominal endurance	12	8	0.6	0.6	**Excluded**	Not common and little evidence supporting its relevance in rugby
Reactive agility	20	0	1	1	Validated	
Tackling	19	1	0.95	0.95	Validated	
Passing for distance	13	7	0.65	0.65	**Excluded**	Not regarded as important as compared to passing for accuracy.
Passing for accuracy	20	0	1	1	Validated	
Catching	19	1	0.95	0.95	Validated	
Kicking for distance	13	7	0.65	0.65	**Excluded**	Position dependent

RHIE-repeated high intensity exercise; LB-lower body; UB-upper body; PHIIRA-prolonged high intensity intermittent running ability; CODS-change of direction speed, results for anthropometric variables are excluded from the table

### Second round

A second round was needed for the experts to review the findings for the first round. In addition, experts had to judge the relevance of muscle flexibility as a characteristic important for inclusion in the test battery, and report on the most commonly assessed muscle for flexibility. The experts had to give an overall impression on whether they agreed or disagreed that the test battery was sufficiently comprehensive in covering all relevant physical or physiological and rugby-specific game skills. Procedurally, this entailed sending or providing a summary of the results of the first round to experts via email or in person. Overall, the proportion of agreement among the experts was calculated as a measure of comprehensiveness of the SCRuM test battery. The relevance of inclusion for muscle flexibility was evaluated as previously described using the CVI calculation method.

#### Results

Out of the 20 experts invited to participate, three (3) international experts timeously responded. Additionally, six (6) local experts were available to participate in the study. Of the 18 variables, RSA was considered not essential for inclusion in the test battery. Experts felt that RSA was already incorporated in the RHIE test. There were also suggestions from three (3) experts for the removal of anaerobic capacity (measured by the Triple 120m shuttle test), since it could be indirectly assessed using the RHIE tests. Muscle flexibility had an I-CVI of 0.89 from eight (8) raters. According to the experts (n = 7), the lower back and hamstring muscle flexibility is commonly assessed in rugby. Overall, all the rugby experts agreed on the comprehensiveness of the test battery in including a wide range of physical or physiological characteristics and rugby-specific skills.

### Part 3: Practical feasibility

This study was conducted to establish the practical feasibility of conducting each of the tests in the logically-validated SCRuM test battery. Literature advocates for the development of test batteries that are feasible, reliable and valid [8, 13, 14, 70]. Feasibility concerns are multifaceted and include assessment of parameters such as equipment needed, cost of equipment, time, procedure, human resources needed, ease of scoring and interpreting test results, safety and duration of the test [8, 13, 14, 70].

### Study design and participants

A cross-sectional descriptive study targeting rugby coaches from high schools and senior rugby clubs in Harare, Zimbabwe was conducted. Coaches were targeted because of their potential in applying the findings of this study in their work environment. For the identification of the rugby coaches, high schools in Harare were categorised into SESRL, CESRL, and IHSRL (Interscholastic High School Rugby Leagues). All the rugby coaches in the SESRL and CESRL were invited to participate by virtue of their schools participating in the reputable leagues. However, a random selection of the schools (n = 48) was conducted for the identification of the coaches in the IHSRL. The league is composed of public and private amateur rugby schools which do not participate in the SESRL or CESRL. All the rugby coaches in the selected schools were then invited to participate. Additionally, senior rugby club coaches were approached on individual basis for possible participation in the study.

### Instrument

A practical feasibility instrument was specifically designed for this study (S1 Appendix). Briefly, Section A elicited demographic data and rugby-related information of the coaches with regards to age, gender, high school or club coaching experience (years started coaching, rugby school team coaches, the league the school is in, any other coaching experience besides school or club rugby) and personal rugby experience (whether they have played rugby in their lifetime, and the level they played). Section B was the feasibility data scoring sheet requesting the coaches to rate the practical feasibility of the each test in the SCRuM test battery as follows: 0-not practically feasible, 1-somewhat feasible, 2-practically feasible. The three main feasibility parameters evaluated included:

1. Test equipment issues: evaluating type of equipment needed and cost of purchasing it.
2. Test procedural issues: evaluating the ease of conducting the ideal procedure and the alternative procedure of the test, duration of test, human personnel needed, and easy of scoring and interpreting test results.
3. Test acceptability issues: evaluating logical acceptability/perceived appropriateness, age-specificity, and safety concerns of the test.

### Procedure

A total of 22 high-schools based in Harare, Zimbabwe were invited to participate. School rugby coaches in these schools willing to participate in the study were recruited. Male head coaches from five (5) senior professional rugby clubs were approached on individual basis and invited to participate in the study. Written informed consent was obtained from the participants. Upon agreeing to participate, the coaches were given the following study documents: the practical feasibility questionnaire and the SCRuM test battery informative document. The latter document had detailed information about each test in the test battery including information on every feasibility parameter from the type of equipment needed, estimated cost of purchasing the equipment, test procedural issues, anticipated duration and human resources needed, ways of scoring and interpreting test results, to information on test acceptability issues.

### Statistical analysis

The 10 feasibility parameters were grouped into three categories based on perceived importance of each parameter:

1. High-Priority Feasibility Parameters (HPFPs): This included equipment needed, procedure of the test, possible modifications to the test or equipment, and cost analysis. These four (4) parameters were considered by the research team as the key determinants of practical feasibility of each test. Participants had to rate the feasibility of each test on the aforementioned four parameters based on: 0-not feasible, 1-somewhat feasible and 2-feasible. The total weighted score for HPFPs was 32 (calculated as the maximum possible feasibility scores for HPFPs, which was 8, multiplied by an arbitrary weighted ratio of 4).
2. Medium-Priority Feasibility Parameters (MPFPs): This included average duration, human resources needed, scoring and interpretation of test scores. These three (3) parameters were considered of moderate importance to practical feasibility. For each of these parameters, each test was rated based on: 0-not feasible, 1-somewhat feasible and 2-feasible. The total weighted score for MPFPs was 12 (calculated as the maximum possible feasibility scores for MPFPs, which is 6, multiplied by an arbitrary weighted ratio of 2 representing moderate importance).
3. Low-Priority Feasibility Parameters (LPFPs): This included age-specificity, logical acceptability and safety. These three (3) parameters were considered of least concern with regards to practical feasibility. For each of these parameters, each test was rated as follows: 0-not feasible, 1-somewhat feasible and 2-feasible. The total weighted score for LPFPs was 6 (calculated as the maximum possible feasibility scores for LPFPs, which is 6, multiplied by a weighted ratio of 1).

The maximum possible weighted total score for the each test item was 50. The calculated average score of each test represented the Test-Feasibility Index (T-FI). T-FI scores were dichotomised for interpretation into high (≥35) and low. Tests with low T-FI warranted complete substitution from the test battery.

### Results

Thirty (30) male junior rugby and senior rugby club coaches volunteered to participate in the study. The mean age of the coaches was 43.6 years (SD = 4.49, age range = 36–56). The total number of years coaching either school or club rugby ranged from 3 to 18 years for the coaches. Of the 17 tests in the SCRuM test battery, the majority (n = 14) were perceived to be practically feasible to be conducted in the local setting (Table 5). Three tests, namely, Reactive Agility Test (RAT), One Repetition Maximum Back Squat (1RM BS) and One Repetition Maximum Bench Press (1RM BP) had average T-FIs below >35. Specifically for the RAT, participants had concerns on the type of equipment needed and the cost of the equipment. For the 1RM BP and 1RM BS tests, feasibility concerns raised were mainly on equipment needed, cost of equipment, age-specificity of the tests and logical acceptability of the tests.

**Table 5:** Practical feasibility results based on coaches assessment of the SCRuM test battery (n = 30).

Construct measured	Corresponding test	Average Feasibility Index Score (T-FI)	Interpretation
Speed	5m, 10m, 20m, 40m speed test	41.9	High practical feasibility
RHIE performance ability	RHIE test	36.5	High practical feasibility
PHIIRA/Endurance	Yo-yo intermittent recovery level 1 test	40.8	High practical feasibility
Anaerobic capacity	Triple 120m shuttle test	42.2	High practical feasibility
CODS/agility	L-run test	43.6	High practical feasibility
LB muscular power	Vertical Jump test	42.7	High practical feasibility
LB muscular strength	1RM back squat test	22.6	**Low practical feasibility**
UB muscular strength	1RM bench press test	23.0	**Low practical feasibility**
UB muscular power	2kg medicine ball chest throw test	43.6	High practical feasibility
Muscle flexibility	Sit-and-Reach test	48.1	High practical feasibility
Reactive agility	Reactive Agility test	32.2	**Low practical feasibility**
Tackling	Tackling proficiency test	41.0	High practical feasibility
Passing for accuracy	Passing for accuracy test	40.1	High practical feasibility
Catching	Running and catching test	40.9	High practical feasibility

T-FI = Test feasibility index, RHIE = repeated high intensity exercise, CODS-change of direction speed, PHIIRA-prolonged high intensity intermittent running ability, 1RM = one repetition maximum.

## Discussion

The present study was conducted to evaluate the logical or content validity and the practical feasibility of each test item in the newly-designed SCRuM test battery developed for use in a large study to determine the anthropometric, physical, physiological and rugby-specific game skills discriminating young male adolescent rugby players by playing abilities and level of competition. Briefly, the test battery had been developed from information gathered through a narrative and systematic literature review [10, 11] combined with results from a qualitative study investigating the perceptions of local rugby coaches of the qualities important in rugby for young adolescent rugby players and the corresponding tests used for evaluation of these qualities. This study was therefore carried out to refine the first version of the SCRuM test battery by evaluating the relevance, comprehensiveness of the test items included in the test battery based on rugby experts perceptions and further ascertain the practical feasibility of conducting the various test items included in the test battery as judged by the local high school coaches likely to be intended users of the test battery.

The primary finding of this present study was that 17 out of the initial 23 variables were considered relevant for inclusion in the SCRuM test battery. This breadth highlights the diversity of physiological or physical qualities and game-specific skills needed by young rugby players between the ages of Under-16 to Under-20 irrespective of position. Another important secondary finding was that proposed tests for upper-and-lower body muscular strength and reactive agility that were included in the first version of the test battery were rather impractical for the Zimbabwean setting and for the age in consideration. These findings suggest need to substitute, or develop new practical feasible tests for the assessment of these important variables.

There was consensus among rugby experts for the inclusion of speed in the SCRuM test battery; a finding confirming the importance of speed in rugby regardless of players position. Speed, which is required for evading opponents, breaking through defensive lines, and scoring tries, has been found to discriminate rugby players of different levels of competition and playing abilities [30, 44, 71–73]. For example, elite junior rugby players were found to have superior sprinting abilities when compared to sub-elite players in a study comparing the physiological and anthropometric characteristics of junior elite and sub-elite rugby players [73]. There is also evidence showing that speed is the most frequently assessed physiological characteristic among rugby players based on findings from a recent systematic review [10]. Motion studies have revealed that rugby players cover varied distances (4m-46m) in a single bout of intense sprinting [25, 32, 33, 43]. This probably accounts for the assessment of speed for over 5m to 60m distances in literature [10]. In the present study, the included speed tests (5m, 10m, 20m, and 40m) were found to be relevant and practically feasible for the Zimbabwean setting and for the intended target population. The tests reflect the speed demands in match play for both the forward and backline players. Shorter sprints (<20m) which assess acceleration ability mainly characterise forwards running and the longer sprints (<40m) assessing maximal velocity commonly observed in game play mainly reflect running distances for the backline players [33, 44, 74].

HIA are an integral component of rugby [20, 25, 27, 75] and therefore the ability to perform RHIE should be an essential requirement. By assessing RHIE, coaches are informed about the level of physical fitness of players for rugby [50]. Concomitantly, the ability to recover quickly from HIA performances (anaerobic capacity) in preparation for a repeated episode should also be important to assess. This probably accounts for experts (n = 3) recommending for the removal of anaerobic capacity as measured by the Triple 120m shuttle run test, since the variable was perceived to be indirectly assessed with the RHIE test. Furthermore, rugby experts selected RHIE for inclusion instead of RSA and REA. Tests for RSA and REA have been challenged for under-estimating the HIA characterising rugby matches [36, 49, 50, 76]. This probably explains the inclusion of specific RHIE tests for both the forward and backline players in the SCRuM test battery. The RHIE test for the forwards had scrummaging episodes as compared to the RHIE test for the backline players, reflecting the importance of the ability to engage in frequent scrummaging for the forward players. However, the proposed RHIE tests showed marginal Test-Feasibility Index (36.5) suggesting possible feasible concerns with the test. The major concerns highlighted included; human personnel needed, time needed to implement the test and age-specific issues considering the specified intensities and durations of RHIE bouts for the test. Rugby experts felt that tests for RHIE probably captured the intensities and durations for professional senior rugby players and may be demanding for the young adolescent high school-children playing rugby. There is need, therefore, to design or adapt the test for junior rugby players to improve the face validity and feasibility of the test among young Zimbabwean rugby players.

There was also consensus for the inclusion of CODS/agility in the test battery. Rugby involves large amounts of acceleration, deceleration and multi-directional running over short distances for all the players regardless of position [17, 39, 44]. This requires rugby players to have good agility without losing balance. Higher agility skills allow rugby players to play in a fast and efficient manner [77]. Therefore, CODS/agility has been reported to be an important variable for rugby players to possess [4, 34, 73, 78–80]. This importance is also evidenced by the frequency of assessment of CODS/agility in rugby players by coaches and sports scientists [10, 81]. In the present study, the proposed test for CODS/agility was found to be relevant and practically feasible for implementation in the Zimbabwean setting. However, the procedural movements of the L-run test were perceived to be “generic” and “mentally rehearseable” leading to better performances. This does not mimic field play, which is characterised largely by unplanned movement patterns [56, 82]. Possibly, it is for this reason that the experts agreed for the inclusion of reactive agility in the test battery. Oorschot et al [11] found that reactive agility was one of the most commonly investigated skill in rugby. In addition, Gabbett et al [18] demonstrated that reactive agility successfully discriminated first grade from second grade rugby players, further suggesting the importance of reactive agility in rugby. However, in the present study, the proposed test for reactive agility had a low practical feasibility index (T-FI = 32.2), indicating possible feasibility challenges with the execution of the test in the Zimbabwean setting. The major areas of concern reported included the equipment needed and the cost of purchasing the equipment. Considering the constraints associated with the assessment of reactive agility, Turner et al [56] recommended use of CODS/agility tests alone for the assessment of agility in soccer players. However, reactive agility seems to be an important variable in rugby as compared to soccer because of the nature of the sport which requires multiple changes of direction in response to stimuli. There is need to incorporate both tests of change of direction speed and reactive agility in protocol development, since episodes of (un)anticipated agile manoeuvres both occur in match play [79]. Nonetheless, there is need for development of new practically feasible tests for the assessment of perceptual or decision-making aspects of agility in young rugby players.

There was perfect agreement among the experts for inclusion of anthropometric assessments (height, mass and skinfold thickness), upper-and-lower muscular power and strength, and muscular flexibility in the test battery. These findings suggest to the importance of these variables in rugby considering the dynamic and physical nature of the sport. Since acquiring professional status in 1995, rugby has grown into a quicker, more dynamic sport with greater emphasis on well-developed physical characteristics of players [81, 83–85]. Rugby players are subjected to frequent and powerful contact situations such as scrummaging, tackling, mauling and rucking [84, 85] which require body mass, muscle flexibility, strength and power. Muscle flexibility optimises eccentric and concentric contraction of the muscle ensuring efficient generation of strength. Leg strength facilitates increased leg drive which assists in sprinting, scrummaging, lifting, and tackling [34]. In addition, muscular strength and power has been reported to reduce the risk of injuries [72]. Performance of game tasks such as kicking, jumping, and lifting also require muscular strength and power generation abilities. Successful teams in international rugby are reported to have had the heaviest and tallest players [30, 86, 87]. Contemporary elite rugby players are known to be physically imposing (bigger and stronger) compared to players of two decades ago [87]. Gabbett [38] found that body mass was an important determinant of selection into a rugby team. Measures of upper-and lower body muscular strength and power were found to discriminate rugby players by level of competition [3, 34, 80]. However, in the present study, proposed tests for lower and upper body strength had low practical feasibility mainly because of the weightlifting restrictions imposed for young high school athletes in the country. Coaches had concerns on a number of feasibility parameters such as the type of equipment needed, the cost of the equipment, safety concerns, and age-specificity of the test with regards to these weightlifting tests. Therefore, there is need to incorporate new tests in the SCRuM test battery for assessment of lower and upper body strength among young rugby players in the Zimbabwean setting.

There was consensus among rugby experts for the inclusion of measures for passing, catching and tackling in the SCRuM test battery. However, measures for kicking were excluded on the basis for being position dependent. These findings suggest to the importance of accuracy in passing, running and catching ability and tackling in the sport of rugby, warranting the inclusion of these skills in screening test batteries. Time motion analysis (TMA) studies identified passing and tackling as key discrete movement activities commonly observed in match play [17, 26, 39]. This is because rugby is a running, passing, catching game with physical collisions such as tackles occurring throughout the entire match [17, 88–90].

### Limitations

The study findings should be interpreted cognisant of number of limitations. The approach used for face and logical validation of the test battery can be criticised due to its potential for subjective and cognitive bias from the experts thereby influencing the validity of the results. However, attempts were made to draw experts from various countries for the different experiences. For the present study, 20 international and local rugby experts were used for this study. Nevertheless, it is possible that the content of the SCRuM test battery could have differed if different experts had been chosen. Achieving appropriate sample size and retaining experts in subsequent rounds was problematic with this study. Of the 63 experts invited, 20 and 9 participants participated in the first and second round, respectively. Therefore, the results reflect the opinions of experts who timeously responded and were willing to participate in the study. Nonetheless, all the experts were recruited based on expertise in the sport of rugby working in various capacities. In addition, literature is controversial on the ideal number of content experts needed in a validation study, but suggestions point between 3 and 10 [68]. This potentially suggests that the sample sizes for the first and second round of the validation may have been appropriate.

Another limitation of the present study was that experts judged the relevance of performance measures for inclusion in the SCRuM test battery based only on anthropometric, physiological characteristics and rugby specific game skills. Due to the complexity of the sport, there are however several other factors, for example, sociological, psychological or perceptual-cognitive skills such as decision making ability, anticipation, tactical awareness which may influence playing performance [72] and may be important to include in test batteries for distinguishing young rugby players. Feasibility study results reflect the opinions and impressions of local rugby coaches used in this particular study considering the contextual resources available at the various schools in Harare, Zimbabwe that were selected. The results could have differed if other schools had been selected or if coaches with different coaching experiences had been used. The coaches used were also coaching at different levels of competition. This accounts for the results on practical feasibility for the upper-and-lower body muscular strength, since they are weight-lifting restrictions in the country depending on the age of the rugby players. Under 16 rugby players are not allowed to weight-lift as compared to the senior U-20 first team rugby players. In addition, the subjective nature of the data gathered may cover major practical feasibility issues which can become apparent during the implementation phase of the study. Therefore, there was need to assess other focus areas of feasibility besides practicality and acceptability issues. Feasibility of the SCRuM test battery could have been assessed in terms of demand (by actually documenting the use of the test battery by coaches in local context) or implementation (the extent, likelihood, and manner in which the SCRuM test battery is fully implemented as planned and proposed) [91]. However, this was not practical given the time limits this study had.

## Conclusion

Rugby is a highly demanding physical and skill-based sport [92]. This is reflected in the component items included as relevant in the SCRuM test battery for profiling young male rugby players which covers a wide range of physical or physiological qualities and skills. Results from face and logical validity studies revealed that the following variables were relevant to be included in the SCRuM test battery: anthropometric qualities (weight, height and skin fold measures), physiological characteristics (speed, RHIE performance ability, PHIIRA/endurance, CODS/agility, anaerobic capacity, upper and lower-body muscular power and strength, muscular flexibility), and rugby-specific game skills (reactive agility, passing for accuracy, tackling proficiency, and catching). The present findings could inform coaches and sports scientists on the relevant attributes, qualities and skills to assess among potential rugby talent. Most of the tests except for upper-and-lower muscular strength and reactive agility were perceived to be practical feasibility to be conducted in Zimbabwean setting. Therefore, there is need to incorporate new tests in the SCRuM test battery for assessment of lower-and-upper body strength and reactive agility in the Zimbabwean setting.

## Supporting information

S1 Table The SCRuM test battery and rationale for inclusion of the tests. (DOCX)

S1 Appendix The practical feasibility questionnaire. (DOCX)
