# Supplementary material for: Logical validation and evaluation of practical feasibility for the SCRuM (School Clinical Rugby Measure) test battery developed for young adolescent rugby players in a resource-constrained environment
Source: PLoS One. 2018 Nov 20;13(11):e0207307. doi: 10.1371/journal.pone.0207307 (PMC6245748; doi:10.1371/journal.pone.0207307)
Supplement: S1 Table — (DOCX) [file pone.0207307.s001.docx]

S1 Table: The SCRuM test battery and rationale for inclusion of the tests

| **SCRuM variables** | **Selection criteria** | | | | | **Test included** | **Reason(s) for inclusion** |
| --- | --- | --- | --- | --- | --- | --- | --- |
| **Variables** | **^a^Test commonly used in the literature** | **Rugby-specific test identified** | **Test commonly used locally** | **^a^Test with sound psychometric properties** | **^a^Level of evidence for the test [59]** |  |  |
| Speed | 10m, 20m, 40m linear speed tests | None | 5m-60m linear speed drills | 5m, 10m, 20m | Limited evidence for test-retest reliability (+) rating for 5m, 10m, 20m speed test;  Limited evidence for construct validity (+) rating for 5m, 10m, 20m speed tests only. | 5m, 10m, 20m, 40m linear speed tests. | 5m, 10m, 20m chosen based on better psychometric properties. 40m speed test incorporated because it is part of speed tests commonly used in the literature and mimics the match demands of longer sprints characteristic of the back players. |
| Repeated sprinting ability (RSA) | Repeated 20m sprint test | Rugby-specific repeated speed (RS^2^) test | *Repeated speed drills for variable distances | None | None | Rugby-specific repeated speed (RS^2^) test. | RS^2^ test was specifically developed for rugby. The test has face validity for assessment of repeated sprinting ability; it mimics the movement patterns (LIA and HIA) of the sport. |
| Repeated effort ability (REA) | REA test | REA test | *Not distinctly assessed. | None | None | Repeated effort ability test | REA test common in the literature; partly rugby specific with tackling efforts in the procedure of repeated sprints. |
|  |  |  |  |  |  |  |  |
| Repeated high intensity exercise (RHIE) performance | RHIE performance test | RHIE performance test | *Not distinctly assessed | None | None | Repeated high intensity exercise (RHIE) performance test | RHIE performance test is commonly used, has face validity for assessment of repeated high intensity activities. |
| Prolonged high intensity intermittent running ability/endurance | Yo-yo intermittent recovery level 1test (Yo-Yo IRT 1) | None | Beep test | None | None | Yo-yo intermittent recovery test level 1 | Yo-Yo IRT 1 is commonly used in literature and in local context for the assessment of the construct of endurance. |
| Maximal aerobic power (MAP) | Multistage fitness (MSF) test | None | None | None | None | Multistage fitness (MSF) test | MSF test commonly used for assessment of MAP. |
| Anaerobic endurance or capacity | Triple 120m shuttle (T120S) test;  Wingate 60 (w60) cycle test;  300m shuttle run test;  400m sprint test (Metabolic Fitness Index for Team Sports) | None | *Not distinctly assessed. | None | None | Triple 120m shuttle (T120S) test | Mimics rugby demands, has face validity for assessing anaerobic capacity as compared to other identified tests such as Wingate 60 (w6o) cycle test, 300m shuttle run test, 400m sprint test. |
|  |  |  |  |  |  |  |  |
| Change of direction speed/agility | 505 test | None | L-run, Illinois agility run test | Modified 505 test  L-run test  505 test | Limited evidence for test-retest reliability of modified 505 test (+) rating (ICC=0.92;%TE=2.5)  Limited evidence on construct validity ( -) rating (ES=0.32)  Limited evidence for test-retest reliability (+) rating (ICC=0.95; %TE=2.8);  Limited evidence on the construct validity (-) rating (ES=0.28)  Limited evidence for test-retest reliability (+) rating (ICC=0.90; %TE=1.9); Limited evidence on the construct validity (-) rating (ES=0.28). | L-run test | L-run test used commonly locally and has fair psychometric properties with relatively high ICC when compared to other agility tests (modified 505 test and 505 test). |
| Lower body muscular power | Countermovement jump (CMJ) test | None | Vertical jump (VJ) test | Vertical jump test | Limited evidence for intra (ICC=0.99) and inter-rater ( ICC=1.00) reliability (+) rating | VJ test | VJ test is locally used by coaches and has better psychometrics compared to other tests for power |
| Lower body muscular strength | One repetition maximum back squat test (1RM BS) | None | U20-back squat  U13-U16-wall sit leg strength, functional squats test | None | None | One repetition maximum back squat test (1RM BS) | 1RM BS is the most commonly used test in the literature. |
| Upper body muscular power | 2kg medicine ball chest throw | None | None | None | None | 2kg medicine ball chest throw | 2kg medicine ball chest throw is commonly used |
| Upper body muscular strength | One repetition maximum bench press (1RM BP) | None | U16-Push up test, flexed arm hang test  U20-bench press | None | None | One repetition maximum bench press (1RM BP) | 1RM BP is commonly assessed in the literature |
| **Upper body muscular endurance | 60s push up test;  60s chin up test;  1RM Bench press repetitions-to-fatigue at 60%;  1RM Bench press repetitions-to-fatigue at 60kg;  1RM Bench press repetitions-to-fatigue at 102.5kg;  Pull up test;  Body mass bench press with repetition; 30s plyometric push-up test | None | Flexed arm hang | BP RTF 60 kg and BP RTF 102.5kg | These two tests had limited evidence on known group validity (+) rating. | Flexed arm hang | Flexed arm hang has a similar procedure as the pull-up test and local coaches are also familiar with its use.  BP RTF 60 kg and BP RTF 102.5kg has better psychometrics but the tests use massive weight load. |
| Abdominal endurance | 60 sit up test | None | Sit ups | None | None | 60 sit up | 60 sit up is commonly used in literature and locally |
| Reactive agility | Reactive agility test (RAT) | None | None | Reactive agility test | Moderate evidence on test-retest reliability for total reactive agility time (++) rating, ICC=0.88, SEM=0.09; ICC=0.82, SEM=0.01.  Moderate evidence on known group validity of the reactive agility test on reactive agility speed (++) ES=1.14, ES=0.73, ES=0.56. | RAT | RAT was chosen because it is commonly used in the literature and has better psychometric properties of test re-test reliability and construct validity |
| Tackling | Tackling proficiency test*** | Tackling proficiency test | Tackling drills | None | None | Tackling proficiency test | Tackling proficiency test chosen because tackling is a skill commonly assessed in the literature. |
| Catching | Running and catching test | Running and catching test | Catching drills | Running and catching pass | Limited evidence on test retest reliability of the running and catching test (-) rating, r=0.53  Limited evidence for known group validity of the running and catching test (w^2^=23.3) + rating | Running and catching test | Running and catching test has better psychometric properties. |
| Kicking | Kicking for distance test | Kicking for distance test | Kicking test | Kicking for distance test | Limited evidence on known group validity of the kicking for distance test (w^2^=29.4; +rating; w^2^=13.9; - rating) | Kicking for distance test | The test has been used in previous studies and has better psychometrics |
| Passing for distance  Passing for accuracy | Passing for distance test  Passing for accuracy 7m test  Passing for accuracy 4m test | Passing for distance test  Passing for accuracy test  Passing for accuracy test | Passing tests  Passing test  Passing test | Passing for distance test  Passing for accuracy test 7m test  Passing for accuracy 4m test | Limited evidence on the test-retest reliability of the passing for distance test (r=0.74); – rating.  Limited evidence on the construct validity of the test (w^2^=32.4), + rating.  Limited evidence on the test-retest reliability for the passing for accuracy 7m test (r=0.66; - rating)  Limited evidence on the known group validity for the test (w^2^=50.7; + rating)  Limited evidence on the test-retest reliability for the test (r=0.39; - rating)  Limited evidence on the known-group validity for the test (w^2^=10.6; - rating) | Passing for distance test  Passing for accuracy 7m test | The test has been used in previous studies and has better psychometric properties.  The test also has better psychometrics |

Anthropometric and body composition measures of height, weight and skin fold measures were omitted from the table; *no specific name mentioned by the local coaches or the characteristic is not distinctly assessed as reported in the literature, LIA-low intensity activities, HIA-high intensity activities; ^a^ represents results presented in a systematic review conducted by Chiwaridzo et al (2017) on the psychometric properties of physiological tests used in rugby and a review conducted by Oorschot et al (2017) on the psychometric properties of game specific skills in rugby; ^§^all scrum variables (physiological characteristics) are named as identified in the literature; ES=effect size ; **all those tests were used in singular studies to measure upper body muscular endurance hence they were taken as all commonly used, *** referred to an umbrella tern used to describe the tackling assessment tests used in the literature for the assessment of tackling; Pearson correlation coefficient, r; BP RTF 60 kg and BP RTF 102.5kg=1RM Bench press repetitions-to-fatigue at 60kg and 1RM Bench press repetitions-to-fatigue at 102.5kg. U-16=Under 16, Under-20=Under 20.
