# Supplementary material for: Logical validation and evaluation of practical feasibility for the SCRuM (School Clinical Rugby Measure) test battery developed for young adolescent rugby players in a resource-constrained environment
Source: PLoS One. 2018 Nov 20;13(11):e0207307. doi: 10.1371/journal.pone.0207307 (PMC6245748; doi:10.1371/journal.pone.0207307)
Supplement: S1 Appendix — (DOCX) [file pone.0207307.s002.docx]

**Practical feasibility study questionnaire for rugby coaches**

Participant Reference number/Code………………... Date……………………………..

*Instructions: Please complete the following questionnaire to the best of your knowledge.*

**Section A: Demographic data and rugby related information**

Age (full years attained): ____

Gender: Male 🞏 Female 🞏

Current School/Club ………………………………………………………………

Type of School Government 🞏 Private 🞏

**High school rugby experience**

1. For how many years in total have you been coaching school rugby/club…………………………………
2. Which year did you start coaching rugby at this particular school/club……………………………………
3. Which school teams have you coached since joining the school (Please tick all that apply): U13 🞏 U14 🞏 U15 🞏 U16 🞏 U17 Second team 🞏 First team🞏.
4. Which high school rugby league does your school participate during the winter rugby games? Super Eight 🞏 Co-educational League 🞏 Interscholastic rugby league 🞏

(*NB: Interscholastic refers to the local rugby competitions schools engage with the neighbouring schools within the same location*)

**Other coaching experience**

1. Do you have any other coaching experience in rugby besides school or club rugby? Yes 🞏 No 🞏
2. If yes, state where else you have coached or still coach besides school/club rugby …………………………………………………………………………………………………………………………………………………………………………………………………………………………

**Personal rugby experience**

1. Have you ever played organised rugby in your lifetime either school or club level? Yes 🞏 No 🞏
2. If yes, at what level did you play? Please tick all that applies.

School 🞏 Local Senior Club 🞏 International Professional Club 🞏 Social/amateur local and international club level 🞏 Zimbabwe National Team 🞏 Other (specify)…………………………………………………………………………………………………..

*School refers to primary and high school rugby, local senior club refers to a senior professional rugby club that played in the Zimbabwe national rugby league, Social refers to amateurish or recreational rugby played for fun and enjoyment in Zimbabwe or abroad. Zimbabwe national team refers to having played for the country.*

1. If yes, for how many years in total did you play rugby as a player at any of the levels you mentioned above? .........................................................................................................................................................

*Include your current experience as well if you are still playing rugby at professional club or a social club*

**Section B: Feasibility data scoring sheet**

1. Please complete the following table on self-perceived feasibility of each item in the SCRuM test battery based on the information provided for each test.

***Instruction:*** *Use the following scoring criteria to rate the practical feasibility of each item in the SCRuM test battery (0=not feasible, 1=somewhat feasible, 2= feasible). Circle the most appropriate response. For logical acceptability, please rate as No (which will be awarded a score of 0), Maybe (which will be awarded a score of 1) and Yes (which will be awarded a score of 2).* *Circle the most appropriate response. Please use the information provided in the information document to help you answer this section.*

| **Test** | **Feasibility parameters** | | | | | | | | | |
| --- | --- | --- | --- | --- | --- | --- | --- | --- | --- | --- |
|  | Equipment | Procedure | Possible modifications | Cost Analysis | Average duration | Human resources needed | Age- specific | Logical acceptable | Scoring and interpretation | Safety |
| Height | 0 1 2 | 0 1 2 | 0 1 2 | 0 1 2 | 0 1 2 | 0 1 2 | 0 1 2 | No Maybe Yes | 0 1 2 | 0 1 2 |
| Weight | 0 1 2 | 0 1 2 | 0 1 2 | 0 1 2 | 0 1 2 | 0 1 2 | 0 1 2 | No Maybe Yes | 0 1 2 | 0 1 2 |
| Skin folds | 0 1 2 | 0 1 2 | 0 1 2 | 0 1 2 | 0 1 2 | 0 1 2 | 0 1 2 | No Maybe Yes | 0 1 2 | 0 1 2 |
| 5m, 10m, 20m, 40m | 0 1 2 | 0 1 2 | 0 1 2 | 0 1 2 | 0 1 2 | 0 1 2 | 0 1 2 | No Maybe Yes | 0 1 2 | 0 1 2 |
| Repeated high intensity exercise performance | 0 1 2 | 0 1 2 | 0 1 2 | 0 1 2 | 0 1 2 | 0 1 2 | 0 1 2 | No Maybe Yes | 0 1 2 | 0 1 2 |
| Yo-Yo Intermittent recovery test | 0 1 2 | 0 1 2 | 0 1 2 | 0 1 2 | 0 1 2 | 0 1 2 | 0 1 2 | No Maybe Yes | 0 1 2 | 0 1 2 |
| Sit and reach test | 0 1 2 | 0 1 2 | 0 1 2 | 0 1 2 | 0 1 2 | 0 1 2 | 0 1 2 | No Maybe Yes | 0 1 2 | 0 1 2 |
| 1RM back squat | 0 1 2 | 0 1 2 | 0 1 2 | 0 1 2 | 0 1 2 | 0 1 2 | 0 1 2 | No Maybe Yes | 0 1 2 | 0 1 2 |
| 1RM bench press | 0 1 2 | 0 1 2 | 0 1 2 | 0 1 2 | 0 1 2 | 0 1 2 | 0 1 2 | No Maybe Yes | 0 1 2 | 0 1 2 |
| L-run test | 0 1 2 | 0 1 2 | 0 1 2 | 0 1 2 | 0 1 2 | 0 1 2 | 0 1 2 | No Maybe Yes | 0 1 2 | 0 1 2 |
| Vertical jump test | 0 1 2 | 0 1 2 | 0 1 2 | 0 1 2 | 0 1 2 | 0 1 2 | 0 1 2 | No Maybe Yes | 0 1 2 | 0 1 2 |
| 2kg medicine ball chest throw test | 0 1 2 | 0 1 2 | 0 1 2 | 0 1 2 | 0 1 2 | 0 1 2 | 0 1 2 | No Maybe Yes | 0 1 2 | 0 1 2 |
| Triple 120m shuttle run | 0 1 2 | 0 1 2 | 0 1 2 | 0 1 2 | 0 1 2 | 0 1 2 | 0 1 2 | No Maybe Yes | 0 1 2 | 0 1 2 |
| Reactive agility test | 0 1 2 | 0 1 2 | 0 1 2 | 0 1 2 | 0 1 2 | 0 1 2 | 0 1 2 | No Maybe Yes | 0 1 2 | 0 1 2 |
| Tackling proficiency test | 0 1 2 | 0 1 2 | 0 1 2 | 0 1 2 | 0 1 2 | 0 1 2 | 0 1 2 | No Maybe Yes | 0 1 2 | 0 1 2 |
| Running and catching test | 0 1 2 | 0 1 2 | 0 1 2 | 0 1 2 | 0 1 2 | 0 1 2 | 0 1 2 | No Maybe Yes | 0 1 2 | 0 1 2 |
| Passing for accuracy test | 0 1 2 | 0 1 2 | 0 1 2 | 0 1 2 | 0 1 2 | 0 1 2 | 0 1 2 | No Maybe Yes | 0 1 2 | 0 1 2 |

1. For each test that scores poor on practical feasibility (**that is a score of 0 and No for logical acceptability**) please indicate below the **areas of most concern** to you for the practical feasibility of the test and if possible provide a recommendation for the concern which may enable the test to be performed feasibly in the local context.

| **Test** | **Areas of concern for the test** | **Tick** | **Other concerns** | **Recommendation for improvement** |
| --- | --- | --- | --- | --- |
| 5m, 10m, 20m, 40m tests | a. A lot of equipment needed |  |  |  |
|  | b. Difficult to execute the testing procedure |  |  |  |
|  | c. Expensive to purchase the equipment |  |  |  |
|  | d. The modifications not changing the practicality of test |  |  |  |
|  | e. Time consuming to perform |  |  |  |
|  | f. Not simple to score and interpret findings |  |  |  |
|  | g. The test invloves a lot of human resources |  |  |  |
|  | h. Safety concerns high |  |  |  |
|  | i. The test not age appropriate for adolescents |  |  |  |
|  | j. The test not logically acceptable or appropriate for rugby |  |  |  |
|  |  |  |  |  |
| Repeated high intensity exercise performance test | a. A lot of equipment needed |  |  |  |
|  | b. Difficult to execute the testing procedure |  |  |  |
|  | c. Expensive to purchase the equipment |  |  |  |
|  | d. modifications not making the test practicable |  |  |  |
|  | e. Time consuming to perform |  |  |  |
|  | f. Not simple to score and interpret findings |  |  |  |
|  | g. The test invloves a lot of human resources |  |  |  |
|  | h. Safety concerns high |  |  |  |
|  | i. The test not age appropriate |  |  |  |
|  | j. The test not logically acceptable or appropriate for rugby |  |  |  |
|  |  |  |  |  |
| Yo-Yo Intermittent recovery test | a. A lot of equipment needed |  |  |  |
|  | b. Difficult to execute the testing procedure |  |  |  |
|  | c. Expensive to purchase the equipment |  |  |  |
|  | d. modifications not making test practical |  |  |  |
|  | e. Time consuming to perform |  |  |  |
|  | f. Not simple to score and interpret findings |  |  |  |
|  | g. The test invloves a lot of human resources |  |  |  |
|  | h. Safety concerns high |  |  |  |
|  | i. The test not age appropriate |  |  |  |
|  | j. The test not logically acceptable or appropriate for rugby |  |  |  |
|  |  |  |  |  |
| L-run | a. A lot of equipment needed |  |  |  |
|  | b. Difficult to execute the testing procedure |  |  |  |
|  | c. Expensive to purchase the equipment |  |  |  |
|  | d. Modifications not making the test practical |  |  |  |
|  | e. Time consuming to perform |  |  |  |
|  | f. Not simple to score and interpret findings |  |  |  |
|  | g. The test invloves a lot of human resources |  |  |  |
|  | h. Safety concerns high |  |  |  |
|  | i. The test not age appropriate |  |  |  |
|  | j. The test not logically acceptable or appropriate for rugby |  |  |  |
|  |  |  |  |  |
| Vertical jump test | a. A lot of equipment needed |  |  |  |
|  | b. Difficult to execute the testing procedure |  |  |  |
|  | c. Expensive to purchase the equipment |  |  |  |
|  | d. Modifications not making the test practical |  |  |  |
|  | e. Time consuming to perform |  |  |  |
|  | f. Not simple to score and interpret findings |  |  |  |
|  | g. The test invloves a lot of human resources |  |  |  |
|  | h. Safety concerns high |  |  |  |
|  | i. The test not age appropriate |  |  |  |
|  | j. The test not logically acceptable or appropriate for rugby |  |  |  |
|  |  |  |  |  |
| 2kg medicine ball chest throw | a. A lot of equipment needed |  |  |  |
|  | b. Difficult to execute the testing procedure |  |  |  |
|  | c. Expensive to purchase the equipment |  |  |  |
|  | d. Modifications not making the test practical |  |  |  |
|  | e. Time consuming to perform |  |  |  |
|  | f. Not simple to score and interpret findings |  |  |  |
|  | g. The test invloves a lot of human resources |  |  |  |
|  | h. Safety concerns high |  |  |  |
|  | i. The test not age appropriate |  |  |  |
|  | j. The test not logically acceptable or appropriate for rugby |  |  |  |
|  |  |  |  |  |
| 1RM bench press | a. A lot of equipment needed |  |  |  |
|  | b. Difficult to execute the testing procedure |  |  |  |
|  | c. Expensive to purchase the equipment |  |  |  |
|  | d. Modifications not making the tests practical |  |  |  |
|  | e. Time consuming to perform |  |  |  |
|  | f. Not simple to score and interpret findings |  |  |  |
|  | g. The test invloves a lot of human resources |  |  |  |
|  | h. Safety concerns high |  |  |  |
|  | i. The test not age appropriate |  |  |  |
|  | j. The test not logically acceptable or appropriate for rugby |  |  |  |
|  |  |  |  |  |
| Sit and reach test | a. A lot of equipment needed |  |  |  |
|  | b. Difficult to execute the testing procedure |  |  |  |
|  | c. Expensive to purchase the equipment |  |  |  |
|  | d. Modifications not making the tests practical |  |  |  |
|  | e. Time consuming to perform |  |  |  |
|  | f. Not simple to score and interpret findings |  |  |  |
|  | g. The test invloves a lot of human resources |  |  |  |
|  | h. Safety concerns high |  |  |  |
|  | i. The test not age appropriate |  |  |  |
|  | j. The test not logically acceptable or appropriate for rugby |  |  |  |
|  |  |  |  |  |
| 1RM back squat | a. A lot of equipment needed |  |  |  |
|  | b. Difficult to execute the testing procedure |  |  |  |
|  | c. Expensive to purchase the equipment |  |  |  |
|  | d. Modfications not making the tests practical |  |  |  |
|  | e. Time consuming to perform |  |  |  |
|  | f. Not simple to score and interpret findings |  |  |  |
|  | g. The test invloves a lot of human resources |  |  |  |
|  | h. Safety concerns high |  |  |  |
|  | i. The test not age appropriate |  |  |  |
|  | j. The test not logically acceptable or appropriate for rugby |  |  |  |
|  |  |  |  |  |
| Reactive agility | a. A lot of equipment needed |  |  |  |
|  | b. Difficult to execute the testing procedure |  |  |  |
|  | c. Expensive to purchase the equipment |  |  |  |
|  | d. Modifications not making the tests practical |  |  |  |
|  | e. Time consuming to perform |  |  |  |
|  | f. Not simple to score and interpret findings |  |  |  |
|  | g. The test invloves a lot of human resources |  |  |  |
|  | h. Safety concerns high |  |  |  |
|  | i. The test not age appropriate |  |  |  |
|  | j. The test not logically acceptable or appropriate for rugby |  |  |  |
|  |  |  |  |  |
| Tackling proficiency test | a. A lot of equipment needed |  |  |  |
|  | b. Difficult to execute the testing procedure |  |  |  |
|  | c. Expensive to purchase the equipment |  |  |  |
|  | d. Modifications not making the tests practical |  |  |  |
|  | e. Time consuming to perform |  |  |  |
|  | f. Not simple to score and interpret findings |  |  |  |
|  | g. The test invloves a lot of human resources |  |  |  |
|  | h. Safety concerns high |  |  |  |
|  | i. The test not age appropriate |  |  |  |
|  | j. The test not logically acceptable or appropriate for rugby |  |  |  |
|  |  |  |  |  |
| Running and Catching test | a. A lot of equipment needed |  |  |  |
|  | b. Difficult to execute the testing procedure |  |  |  |
|  | c. Expensive to purchase the equipment |  |  |  |
|  | d. Modifications not making the tests practical |  |  |  |
|  | e. Time consuming to perform |  |  |  |
|  | f. Not simple to score and interpret findings |  |  |  |
|  | g. The test invloves a lot of human resources |  |  |  |
|  | h. Safety concerns high |  |  |  |
|  | i. The test not age appropriate |  |  |  |
|  | j. The test not logically acceptable or appropriate for rugby |  |  |  |
|  |  |  |  |  |
| Passing for accuracy test | a. A lot of equipment needed |  |  |  |
|  | b. Difficult to execute the testing procedure |  |  |  |
|  | c. Expensive to purchase the equipment |  |  |  |
|  | d. Modificatios not making the tests practical |  |  |  |
|  | e. Time consuming to perform |  |  |  |
|  | f. Not simple to score and interpret findings |  |  |  |
|  | g. The test invloves a lot of human resources |  |  |  |
|  | h. Safety concerns high |  |  |  |
|  | i. The test not age appropriate |  |  |  |
|  | j. The test not logically acceptable or appropriate for rugby |  |  |  |
|  |  |  |  |  |
| Triple 120m shuttle run test | a. A lot of equipment needed |  |  |  |
|  | b. Difficult to execute the testing procedure |  |  |  |
|  | c. Expensive to purchase the equipment |  |  |  |
|  | d. Modifications not making the tests practical |  |  |  |
|  | e. Time consuming to perform |  |  |  |
|  | f. Not simple to score and interpret findings |  |  |  |
|  | g. The test invloves a lot of human resources |  |  |  |
|  | h. Safety concerns high |  |  |  |
|  | i. The test not age appropriate |  |  |  |
|  | j. The test not logically acceptable or appropriate for rugby |  |  |  |
|  |  |  |  |  |
| Weight | a. A lot of equipment needed |  |  |  |
|  | b. Difficult to execute the testing procedure |  |  |  |
|  | c. Expensive to purchase the equipment |  |  |  |
|  | d. Modifications not making the tests practical |  |  |  |
|  | e. Time consuming to perform |  |  |  |
|  | f. Not simple to score and interpret findings |  |  |  |
|  | g. The test invloves a lot of human resources |  |  |  |
|  | h. Safety concerns high |  |  |  |
|  | i. The test not age appropriate |  |  |  |
|  | j. The test not logically acceptable or appropriate for rugby |  |  |  |
|  |  |  |  |  |
| Height | a. A lot of equipment needed |  |  |  |
|  | b. Difficult to execute the testing procedure |  |  |  |
|  | c. Expensive to purchase the equipment |  |  |  |
|  | d. Modifications not making the tests practical |  |  |  |
|  | e. Time consuming to perform |  |  |  |
|  | f. Not simple to score and interpret findings |  |  |  |
|  | g. The test invloves a lot of human resources |  |  |  |
|  | h. Safety concerns high |  |  |  |
|  | i. The test not age appropriate |  |  |  |
|  | j. The test not logically acceptable or appropriate for rugby |  |  |  |
|  |  |  |  |  |
| Skinfolds measures | a. A lot of equipment needed |  |  |  |
|  | b. Difficult to execute the testing procedure |  |  |  |
|  | c. Expensive to purchase the equipment |  |  |  |
|  | d. Modifications not making the test practical |  |  |  |
|  | e. Time consuming to perform |  |  |  |
|  | f. Not simple to score and interpret findings |  |  |  |
|  | g. The test invloves a lot of human resources |  |  |  |
|  | h. Safety concerns high |  |  |  |
|  | i. The test not age appropriate |  |  |  |
|  | j. The test not logically acceptable or appropriate for rugby |  |  |  |
|  |  |  |  |  |
